# Structural and functional remodeling of the atrioventricular node with aging in rats: The role of hyperpolarization-activated cyclic nucleotide–gated and ryanodine 2 channels

**DOI:** 10.1016/j.hrthm.2017.12.027

**Published:** 2018-05

**Authors:** Yawer Saeed, Ian P. Temple, Zoltan Borbas, Andrew Atkinson, Joseph Yanni, Michal Maczewski, Urszula Mackiewicz, Mariam Aly, Sunil Jit R.J. Logantha, Clifford J. Garratt, Halina Dobrzynski

**Affiliations:** ∗ Institute of Cardiovascular Sciences, University of Manchester, Manchester, United Kingdom; † Manchester Heart Centre, Central Manchester University Hospitals NHS Trust, Manchester, United Kingdom; ‡ Department of Clinical Physiology, Centre of Postgraduate Medical Education, Warsaw, Poland

**Keywords:** Aging, AV node, I_f_, Ion channel expression, Ryanodine

## Abstract

**Background:**

Aging is associated with an increased incidence of atrioventricular nodal (AVN) dysfunction.

**Objective:**

The aim of this study was to investigate the structural and functional remodeling in the atrioventricular junction (AVJ) with aging.

**Methods:**

Electrophysiology, histology, and immunohistochemistry experiments on male Wistar Hannover rats aged 3 months (n = 24) and 2 years (n = 15) were performed. Atrio-His (AH) interval, Wenkebach cycle length (WBCL), and AVN effective refractory period (AVNERP) were measured. Cesium (2 mM) was used to block hyperpolarization-activated cyclic nucleotide–gated (HCN) channels, while ryanodine (2 μM) was used to block ryanodine 2 (RyR2) channels. Protein expression from different regions of the AVJ was studied using immunofluorescence. The expression of connexins (connexin 43 and connexin 40), ion channels (Hyperpolarization-activated cyclic nucleotide-gated channel 4 (HCN4), voltage sensitive sodium channel (Na_v_1.5), and L-Type calcium channel (Ca_v_1.3)), and calcium handling proteins (RyR2 and sarco/endoplasmic reticulum calcium ATPaset type 2a (SERCA2a)) were measured. Morphological characteristics were studied with histology.

**Results:**

Without drugs to block HCN and RyR2 channels, there was prolongation of the AH interval, WBCL, and AVNERP (*P* < .05) with aging. In young rats only, cesium prolonged the AH interval, WBCL, and AVNERP (*P* < .01). Ryanodine prolonged the AH interval and WBCL (*P* < .01) in both young and old rats. Immunofluorescence revealed that with aging, connexin 43, HCN4, Na_v_1.5, and RyR2 downregulate in the regions of the AVJ and connexin 40, SERCA2a, and Ca_v_1.3 upregulate (*P* < .05). Aging results in cellular hypertrophy, loosely packed cells, a decrease in the number of nuclei, and an increase in collagen content.

**Conclusion:**

Heterogeneous ion channel expression changes were observed in the AVJ with aging. For the first time, we have shown that HCN and RyR2 play an important role in AVN dysfunction with aging.

## Introduction

**Supplemental Figure 1:**
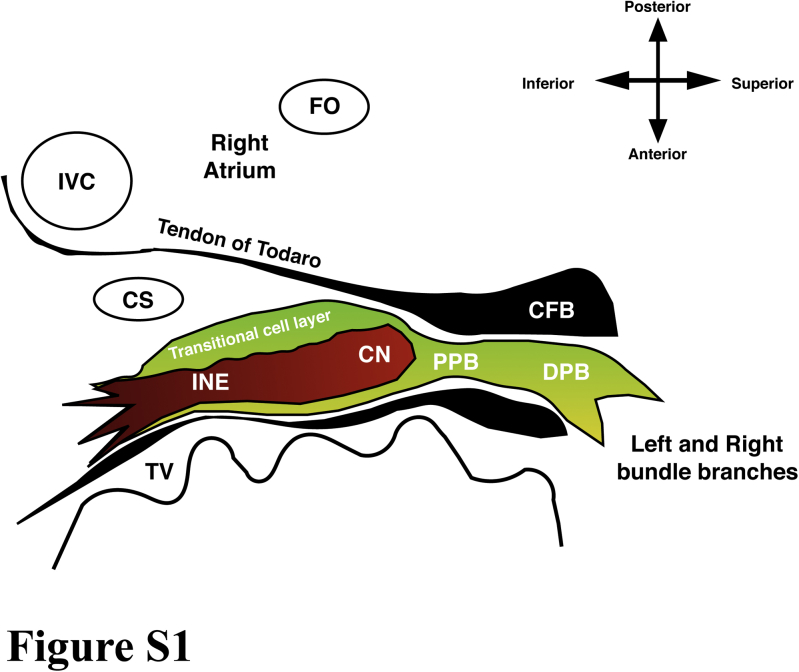
Schematic drawing showing the regions of the rat atrioventricular junction (AVJ). Also shown is the transitional cell layer. CS (coronary sinus), CFB (Central fibrous body) INE (Inferior nodal extension), CN (Compact node), Proximal penetrating bundle (PPB), Distal penetrating bundle (DPB), IVC (Inferior Vena cava), FO (Foramen ovale), TV (Tricuspid Valve).

The atrioventricular node (AVN), since its discovery by Sunao Tawara,^1^ has held scientists and clinicians interested in its structure and function. The structure of the AVN is intricate, which also underlies the complex functional characteristics of AVN conduction. The structure of the atrioventricular junction (AVJ) includes the inferior nodal extension (INE), compact node (CN), proximal penetrating bundle (PPB), and distal penetrating bundle or His bundle (DPB/His)^2^ (Supplemental Figure S1).

AVN conduction has the unique characteristic of “delay” or slow conduction. In the AVJ, 9 action potential (AP) recordings have been made, which show the functional complexity of the AVJ.^3^ The cellular mechanisms that influence AVN conduction are still subject to debate. Some studies have proven the absence of a Na^+^ current (I_Na_) and confirmed the presence of a funny current (I_f_) in the “N cells,” which is found in the CN of a rabbit's heart. These N cells are also dependent on calcium currents for the AP generation. Thus, the AP in N cells is characterized by a small amplitude and a slow rate of rise of the upstroke.^4^, ^5^ I_f_ is the hyperpolarization-activated current carried by hyperpolarization-activated cyclic nucleotide–gated (HCN) channels and is involved in phase 4 of the AP in the pacemaker cells.^6^ The oscillatory release of Ca^2+^ from the sarcoplasmic reticulum (SR) has also been shown to be an equally important phenomenon in AP generation in the pacemaker cells.^7^

AVN function declines with age, which is observed in multiple studies in humans, rabbits, and rats.^8^, ^9^, ^10^ The electrophysiological measurements show prolonged atrioventricular (AV) conduction time, PR/PQ interval, atrio-His (AH) interval, His-ventricular interval, Wenkebach cycle length (WBCL), and atrioventricular nodal effective refractory period (AVNERP). Although aging studies on the cardiac conduction system have been performed, the explanation of AVN dysfunction with aging has not yet been established in terms of ion channel expression. Aging certainly has a part to play in the pathophysiology of AV block, as the incidence is higher in the elderly population.^11^

In the present study, we have investigated the role of I_f_ and ryanodine 2 (RyR2)–mediated calcium release from the SR in AVN conduction with aging. The electrophysiological experiments in young and old rats were performed with cesium (I_f_ blocker) and ryanodine (functional blocker of RyR2 channels). Furthermore, the expression of ion channels, connexins, and calcium handling proteins in the AVJ were studied by immunohistochemistry. Morphological changes were studied with histology.

## Methods

Materials and methods are described in detail in the Supplement. Male Wistar Hannover rats (young, n = 24; old, n = 15) were used in this study. All animal procedures were performed in accordance with the UK Animals (Scientific Procedure) Act 1986 and approved by the University of Manchester. Electrophysiological experiments were carried out on the AVN preparations in a tissue bath with oxygenated Tyrode's solution.^12^ The spontaneous sinus node (SN) cycle length (SCL), paced AH interval, WBCL, AVNERP, and AVN functional refractory period (AVFRP) were measured using bipolar electrodes. Measurements were then repeated with cesium (2 mM, specific for I_f_) and ryanodine (2 μM, specific for RyR).^13^, ^14^

Tissue sections (20 μm) were stained with Masson's trichome in order to study the cellular architecture. Picrosirius red stain was used for collagen signal estimation. Immunohistochemistry was carried out using established methods, as described previously.^15^ A summary of antibodies is presented in Supplemental Table S1. These antibodies have been successfully used before by our group.^16^, ^17^

Prism 6 (GraphPad software, La Jolla, CA) for Mac has been used for data entry and statistical analysis. The Student *t* test was used to compare mean ± standard error of the mean values and compute *P* values and 95% confidence intervals (CIs). The paired *t* test was performed on experiments involving pre- and postmeasurements with cesium and ryanodine. A *P* value of ≤.05 is considered statistically significant.

## Results

### Electrophysiological experiments on young and old hearts without drugs

The comparison between young (n = 14) and old (n = 6) hearts without drugs showed that SCL, AH interval, WBCL, AVNERP, and AVFRP all prolonged significantly with aging (Table 1). Electrophysiological and immunohistochemistry experiments are conducted on separate hearts.

**Table 1:** Changes in electrophysiological measurements with aging

Variable	SCL (ms)	AH interval (ms)	WBCL (ms)	AVNERP (ms)	AVFRP (ms)
Young heart (n = 14)	259.10 ± 12.3	41.33 ± 3.60	148.70 ± 6.32	103.20 ± 7.16	141.10 ± 8.87
Old heart (n = 6)	370.50 ± 23.10	63.60 ± 5.144	216.80 ± 31.44	168.30 ± 6.811	184.80 ± 8.2
*P*	.0002	.0049	.0038	.0002	.01
95% CI	58.19–164.7	7.65–36.88	25.72–110.4	39.03–91.03	10.7–75.01

AH = Atrio-His; AVFRP = atrioventricular nodal functional refractory period; AVNERP = atrioventricular nodal effective refractory period; CI = confidence interval; SCL = sinus node cycle length; WBCL = Wenkebach cycle length.

### Electrophysiological experiments with cesium and ryanodine

**Figure 1:**
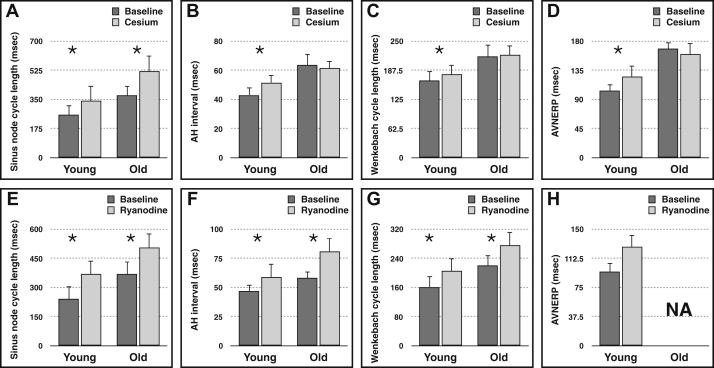
(**A**-**H**) Changes in electrophysiological measurements in young (n = 14) and old (n = 6) hearts' atrioventricular nodal preparation with and without administration of drugs. The data indicate the changes with cesium (**A**-**D**) and ryanodine (**E**-**H**). The AVNERP measurement was not possible in old hearts with ryanodine because of the Wenkebach phenomenon with the S1 drive train. AH = atrio-His; AVNERP = atrioventricular nodal effective refractory period. ∗ *P* < .05.

Figure 1 and Supplemental Figure S2 show the changes in SCL, AH interval, WBCL, and AVNERP after the application of drugs. First, 2 mM cesium was used to block I_f_ . Cesium results in significant prolongation of the spontaneous SCL in both young and old rat hearts. In the young hearts, Cs^+^ prolonged SCL by 32% from 259.1 ± 12.33 to 342.8 ± 21.6 ms (95% CI 61.116–135.0 ms; *P* < .005). In the old hearts, Cs^+^ prolonged SCL by 40% from 374 ± 27.96 to 522 ± 70.17 ms (95% CI 12.40–283.6 ms; *P* < .05). The effect on AV conduction is markedly different in the young and old hearts. In the young hearts, Cs^+^ prolonged AH interval by 20% from 43.1 ± 3.62 to 51.67 ± 5.144 ms (95% CI 2.38–12.28 ms; *P* < .05), whereas in the old hearts the change is only of 2% from 63.60 ± 5.14 to 61.40 ± 4.844 ms (*P* = .48). Similarly, in the young hearts, Cs^+^ prolonged WBCL by 8% from 165.9 ± 10.58 to 179.3 ± 16.03 ms (95% CI 3.22–40.93 ms; *P* < .05), whereas in the old hearts, WBCL changes from 216.8 ± 31.44 to 220.0 ± 30.28 ms (*P* = .63). In the young hearts, Cs^+^ prolonged AVNERP by 20% from 103.3 ± 6.40 to 124.9 ± 10.68 ms (95% CI 4.018–39.18 ms; *P* < .05), whereas in old hearts, AVNERP changes from 168.3 ± 6.811 to 159.8 ± 6.35 ms (*P* = .16).

The effect of ryanodine is equally interesting. Ryanodine prolonged SCL in the young hearts by 53% from 241.1 ± 9.63 to 369.8 ± 25.4 ms (95% CI 65.38–141.1 ms; *P* < .005), whereas in the old hearts it prolonged SCL by 38% from 368.4 ± 25.81 to 507.8 ± 75.52 ms (95% CI 1.31–275.9 ms; *P* < .05). In the young hearts, ryanodine prolonged AH interval by 25% from 47.00 ± 6.08 to 59.00 ± 7.72 ms (95% CI 5.35–28.93 ms; *P* < .05) and WBCL by 27% from 161.5 ± 10.58 to 205.1 ± 22.71 ms (95% CI 13.86–105.4 ms; *P* < .05), whereas in the old hearts, ryanodine prolonged AH interval by 39% from 58.20 ± 3.30 to 81.11 ± 6.61 ms (95% CI 8.87–36.72 ms; *P* < .05) and WBCL by 25% from 220.08 ± 32.24 to 276.8 ± 38.72 ms (95% CI 11.77–100.2 ms; *P* < .05). AVNERP measurement was not possible with ryanodine as the 200-ms S1 drive train results in the Wenkebach phenomenon in the majority of ryanodine-treated hearts. No interaction between the drug and a particular age group is seen.

### Changes in the size of the AVJ, cellular architecture, fibrosis, and cell size with aging

**Supplemental Figure 3:**
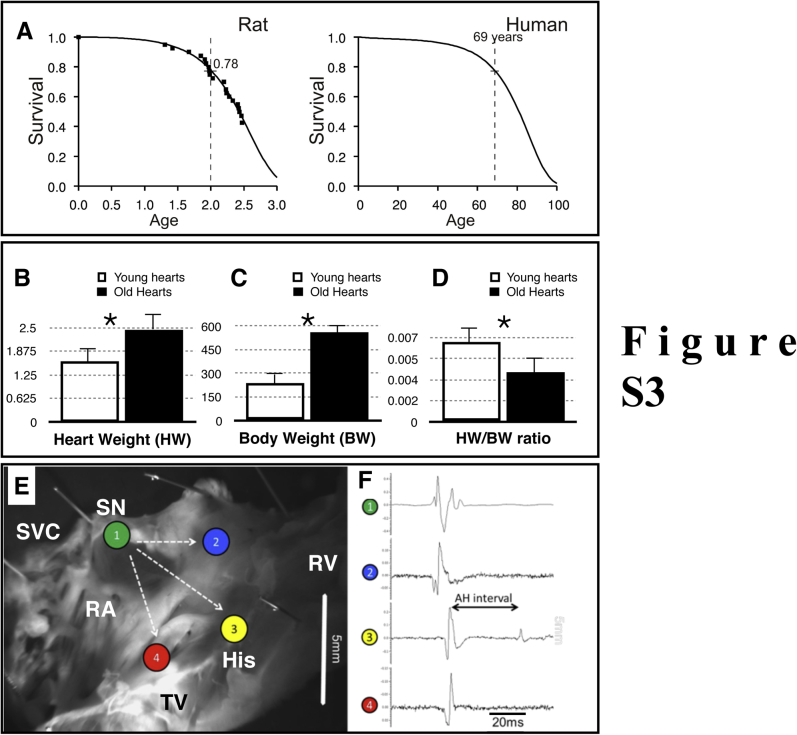
(A) Survival curves in rats and humans. (B, C, D) Heart weight, body weight and heart weight/body weight ratio in young (n=6) and old rats (n=6). * shows p value <0.05. (E,F) Bipolar recording from the AV node preparation. (E) Photograph of preparation. Once the His bundle had been identified the other three electrodes were positioned. Electrode 1 was positioned at the site of earliest activation (the SN). Electrode 2 was positioned at the atrial septum, electrode 3 was at the His electrode and electrode 4 was positioned between the coronary sinus and the tricuspid valve annulus. (F) Bipolar recordings from the 4 electrodes marked in E. Earliest activation was at electrode 1 and latest at electrode 4. This represents rapid atrial activation spreading from the SA node (marked with white dashed arrows). At the His signal (electrode 3) there was a discrete pause which was followed by a second smaller sharp deflection. This second deflection is the His signal. The time between the atrial and His signal is the AH interval. Adapted from Temple.^12^

The older AVJ regions are larger as shown by 3-dimensional measurements. Statistical differences are seen in the height (vertical axis) and volume of the AVJ regions (Supplemental Table S2). The comparison between body weight, heart weight, and heart weight/body weight ratio is shown in Supplemental Figure S3.

**Figure 2:**
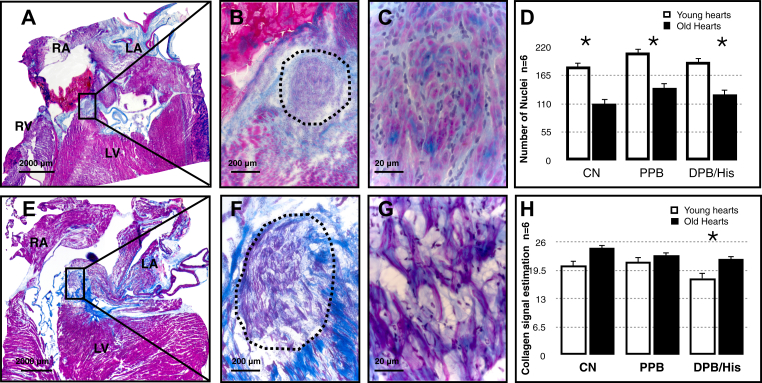
Masson's trichome staining. Proximal penetrating bundle (PPB) in young and old hearts. **A**–**C:** Sections at the PPB level at different magnifications in a young heart. **E**–**G:** Comparable images in an old heart. PPB is marked with a black dotted line. Pink/purple indicates myocytes and black blue indicates nuclei in high-magnification images (panels **C** and **G**). LA = left atrium; LV = left ventricle; RA = right atrium; RV = right ventricle. Bar is shown in each image. Red seen in panel **A** indicates clotted blood. Panels **C** and **G** showed disruption in cellular architecture seen with aging. **D:** Number of nuclei measured in high-magnification images in the regions of the atrioventricular junction. **H:** Collagen signal estimation in young and old hearts' atrioventricular junction measured by Picrosirius red staining and polarized microscopy. CN = compact node; DPB/His = distal penetrating bundle or His bundle; PPB = proximal penetrating bundle. Young hearts, n = 6; old hearts, n = 6. ∗ *P* < .05.

**Supplemental Figure 4:**
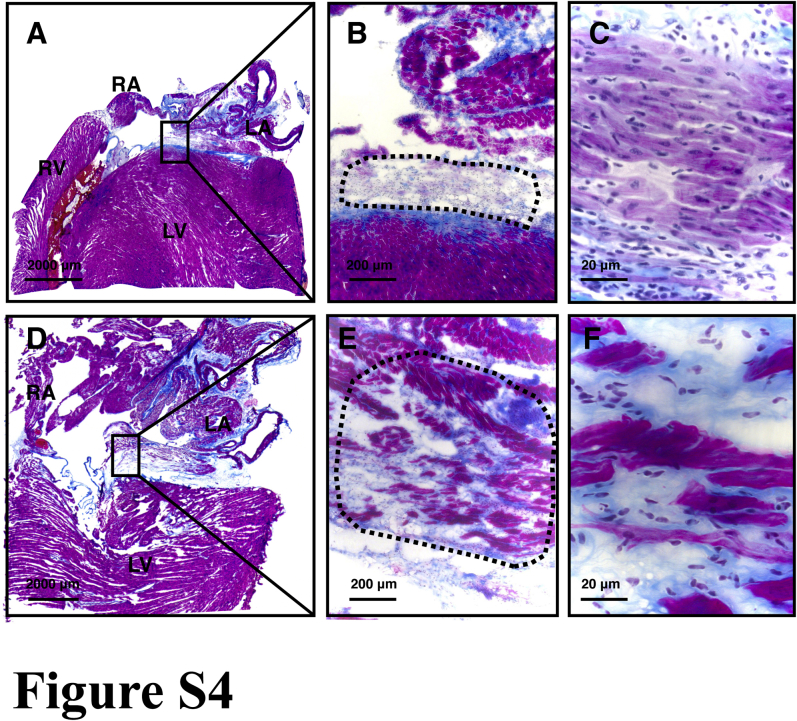
**Masson’s trichome. Young and old inferior nodal extension (INE). The** top panel (A,B,C) show sections at INE level at different magnifications in a young heart and bottom panel (D,E,F) shows the comparable images in old heart at INE level INE is marked with a black dotted line. Myocytes are pink/purple and nuclei are black blue in high magnification images (C, F). Left atrium (LA), right atrium (RA), left ventricle (LV), right ventricle (RV). Bar is shown in each image. The red colour seen in A and D is clotted blood.

Masson's trichome stain showed cellular disarray in older myocytes that are loosely packed and more irregularly arranged in all regions of the AVJ (Figure 2 and Supplemental Figure S4). The number of nuclei reduced with aging in the CN, PPB, and DPB (Figure 2). The nuclei were counted for each rat heart by using high-magnification images.

**Figure 3:**
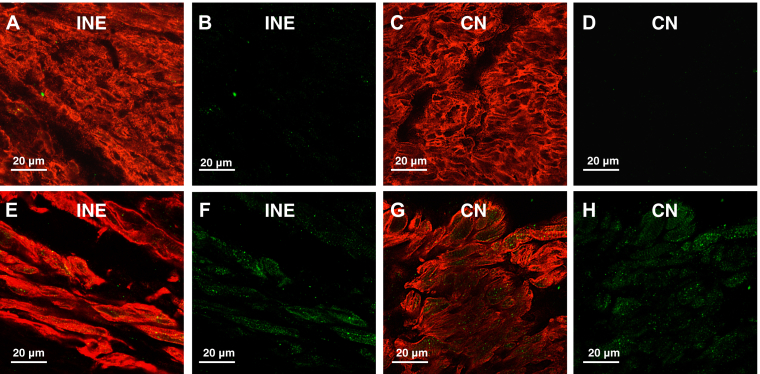
High-magnification confocal microscopy images of the AVJ with Cx40 (green) and Cav3 (red) immunolabeling. **A**–**D:** AVJ components in a young heart. **E**–**H:** AVJ components in an old heart. Cx40 expression is shown separately in panels **B**, **D**, **F**, and **H** to clearly illustrate Cx40 expression as well as its location within the myocardial cells. Bar = 20 μm. Young hearts, n = 6; old hearts, n = 6. AVJ = atrioventricular junction; Cav3 = caveolin-3; CN = compact node; Cx40 = connexin 40; INE = inferior nodal extension.

**Figure 4:**
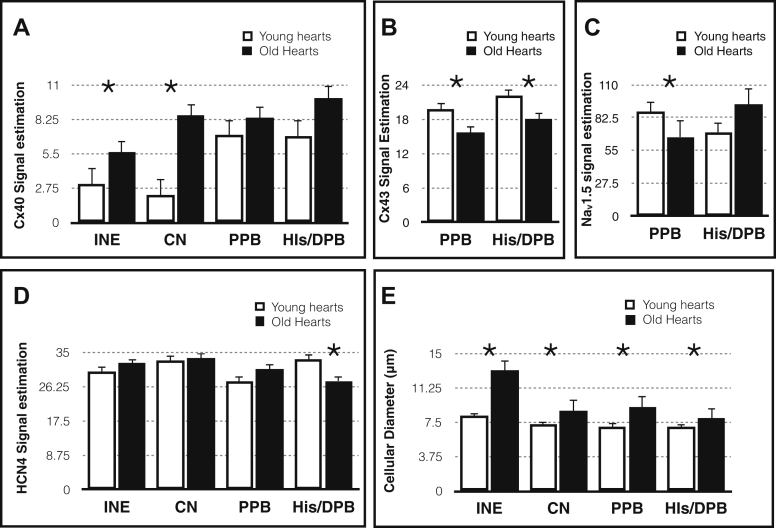
Changes in the expression of Cx40 (**A**), Cx43 (**B**), Na_v_1.5 (**C**), HCN4 (**D**), and cellular diameter (**E**) of the myocardial cells in the atrioventricular junction. Cellular diameter is measured in a high-magnification image in at least 20 myocardial cells in each heart. Values are presented as mean ± standard error of the mean. Young hearts, n = 6; old hearts, n = 6. ^∗^*P* < .05. CN = compact node; Cx40 = connexin 40; Cx43 = connexin 43; DPB/His = distal penetrating bundle or His bundle; HCN4 = Hyperpolarization-activated cyclic nucleotide-gated channel 4; INE = inferior nodal extension; Na_v_1.5 = Voltage sensitive sodium channel; PPB = proximal penetrating bundle.

**Supplemental Figure 5:**
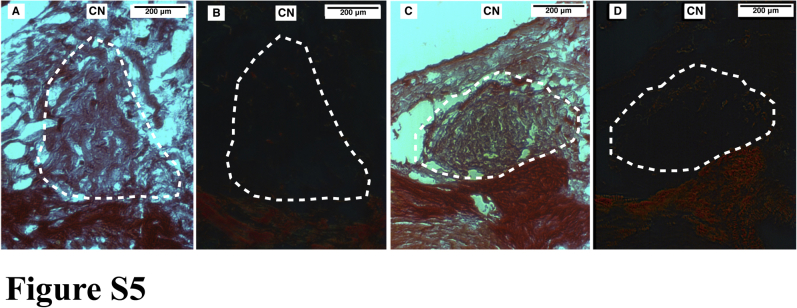
**Picrosirius red stain. Images of the young and old compact node (CN) with light and polarized microscope. (**A) A young heart bright field image, (B) same image taken with polarized microscope. (C) The old CN bright field microscope image, (D) same image taken with polarized microscope. Red and green bisference can be easily seen with a dark background; red bisference represent thick collagen fibers, green bisference represent thin collagen fibers. The CN is marked with a white dotted line. Bar is shown in each image.

**Supplemental Figure 6:**
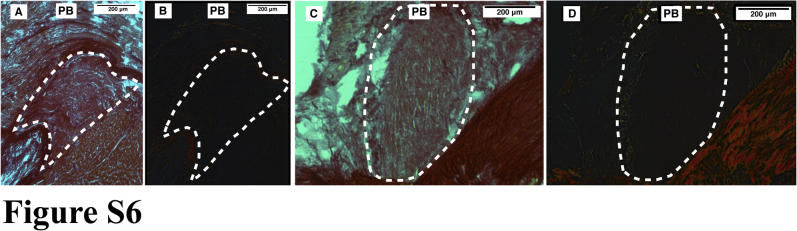
**Picrosirius red stain: Images of the young and old proximal penetrating Bundle (PPB). (**A) young PPB bright field image, (B) same image taken with polarized microscope. (C) old PB bright field image, (D) same image taken with polarized microscope. Red and green bisference represent thick and thin collagen fibers respectively. The PPB is marked with a white dotted line. Old PPB shows high green bisference.

**Supplemental Figure 7:**
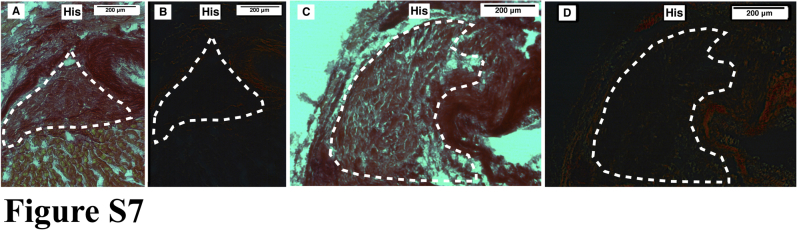
**Picrosirius red stain: Images of young and old DPB or His Bundle (His).** (A) A Young His bright field image, (B) same image taken with polarized microscope. (C) old His bright field image, (D) same image taken with polarized microscope. Red and green bisference represent thick and thin collagen fibers respectively. The His is marked with a white dotted line.

**Supplemental Figure 8:**
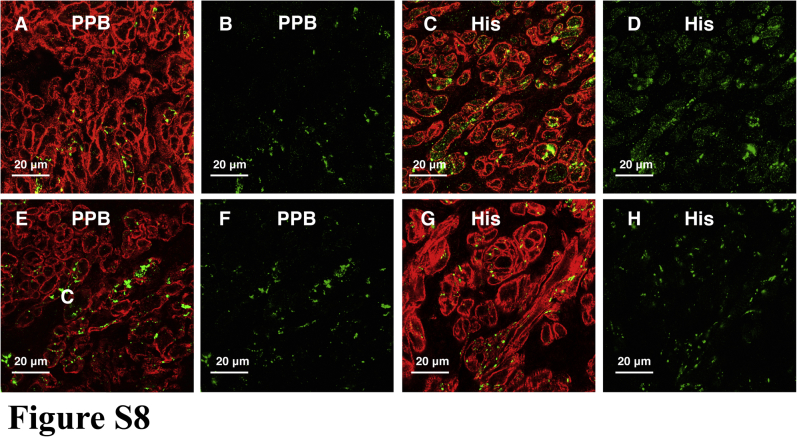
Confocal microscope high magnification images of the AVJ with Cx40 (green) and Cav3 (red) immunolabelling. Proximal penetrating bundle (PPB), distal penetrating bundle/His bundle (His). Top panel shows the AVJ components in young hearts and bottom panel shows the AVJ components in old hearts. Bar=20μm.

The total collagen content increased with aging in the DPB; this was assessed by Picrosirius red staining and polarized microscopy. The increasing trend in total collagen content was also observed in the CN and PPB (Figure 2 and Supplemental Figures S5–S7). Hypertrophy is observed in all regions of the AVJ. The cell diameter (at least 20 cells measured in each region) was estimated by immunolabeling for the membrane marker caveolin-3, a structural protein found in the caveolae of myocytes (Figures 3 and 4 and Supplemental Figure S8).

### Changes in expression of ion channels, calcium handling proteins, and gap junctions with aging

#### Age-dependent change in the expression of connexins, HCN4, and Na_v_1.5

**Figure 5:**
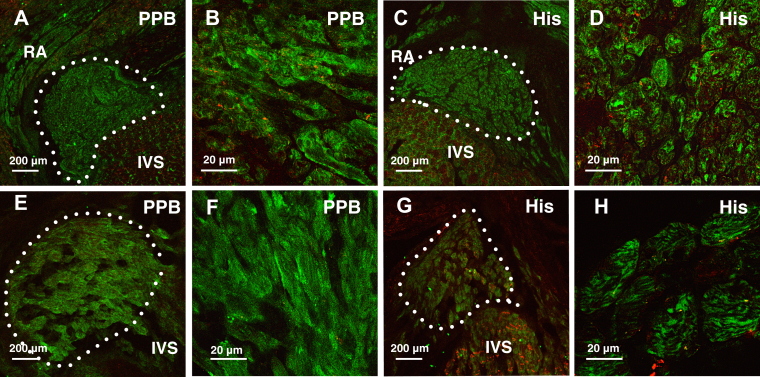
**A**–**D:** AVJ regions in a young heart. **E**–**H:** AVJ regions in an old heart. Panels **A**, **C**, **E**, and **G** show low-magnification confocal microscopy images of the AVJ with HCN4 (green) and Cx43 (red) immunolabeling. Panels **B**, **D**, **F**, and **H** show the corresponding high-magnification images in the PPB and His. Bar is shown in each image. Young hearts, n = 6; old hearts, n = 6. AVJ = atrioventricular junction; Cx43 = connexin 43; HCN4 = Hyperpolarization-activated cyclic nucleotide-gated channel 4; His = His bundle; IVS = interventricular septum; PPB = proximal penetrating bundle.

Connexin 40 (Cx40) expression increased with aging in the INE by 82% (95% CI 0.67–4.39; *P* < .01) and in the CN by 289% (95% CI 2.42–10.45; *P* < .005). There is an upregulation trend seen in the PPB and DPB (Figures 4 and 5 and Supplemental Figure S8). Connexin 43 (Cx43) expression decreased in the PPB by 21% (95% CI −6.87 to −1.31; *P* < .005) and DPB by 20% (95% CI −7.57 to −0.49; *P* < .05) (Figures 4 and 5).

**Supplemental Figure 9:**
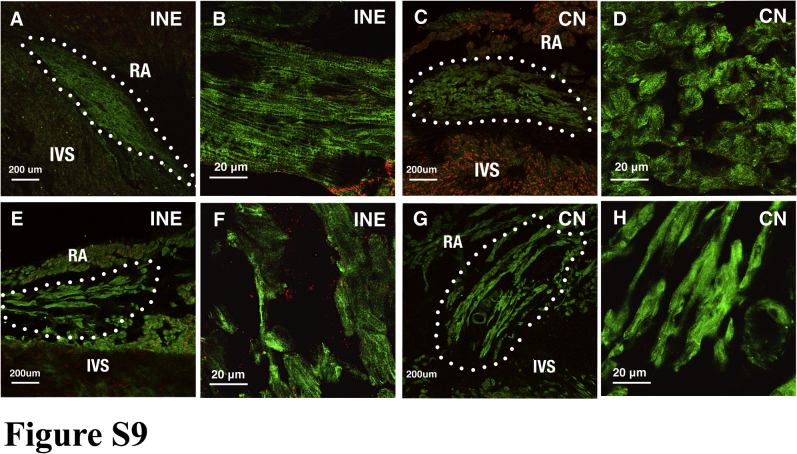
Confocal microscope images, top panel shows the AVJ components in young hearts and bottom panel shows the AVJ components in old hearts. **A, C, E, G:** Low magnification images of the AVJ with HCN4 (green) and Cx43 (red) immunolabelling. Poor Cx43 expression is shown in the INE and CN. **B, D, F, H:** High magnification images of the INE and CN. The Cx43 expression in the right lower corner of image B is part of the right atrium. Inferior nodal extension (INE), compact node (CN).

Hyperpolarization-activated cyclic nucleotide-gated channel 4 (HCN4) expression decreased with aging in the DPB/His by 17% (95% CI −11.6 to 0.03; *P* < .05). Interestingly, although the results are not significant in the CN, INE, and PPB, the trend is of upregulation with aging (Figures 4 and 5 and Supplemental Figure S9).

**Supplemental Figure 10:**
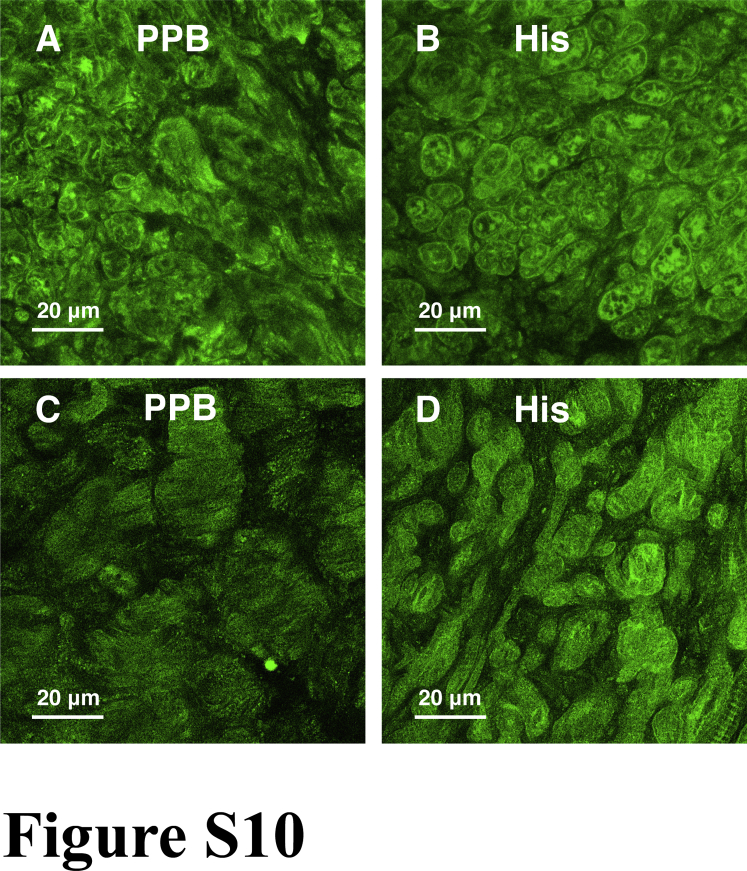
Confocal microscope high magnification images of the AVJ with Nav1.5 (green) immunolabelling, proximal penetrating bundle (PPB), distal penetrating bundle/His bundle (His). Top panel shows the AVJ components in young hearts and bottom panel in old hearts. Bar=20μm.

Voltage sensitive sodium channel (Na_v_1.5) expression tends to decrease with aging in the CN by 27% (95% CI −47.79 to −3.74; *P* < .05) and in the PPB by 24% (95% CI −38.46 to −3.76; *P* < .05) (Figure 4 and Supplemental Figure S10).

#### Age-dependent changes in the expression of calcium handling proteins and Ca_v_1.3

**Figure 6:**
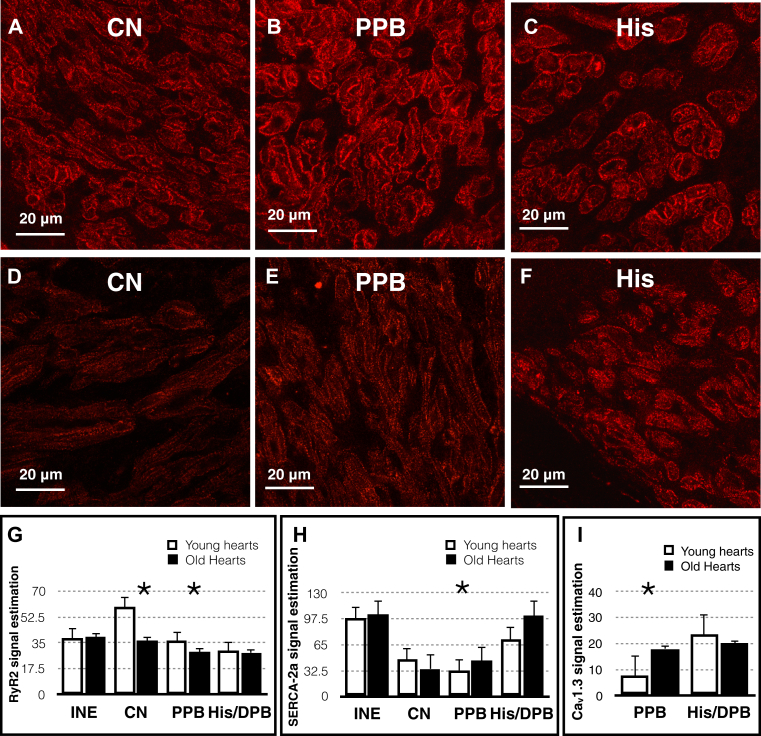
High-magnification confocal microscopy images of the AVJ with RyR2 (red) immunolabeling. **A**–**C:** AVJ regions in a young heart. **D**–**F:** AVJ regions in an old heart. Bar = 20 μm. **G**–**H:** Changes in RyR2, SERCA2a, and Ca_v_1.3 expression with aging. Values are presented as mean ± standard error of the mean. Young hearts, n = 6; old hearts, n = 6. ^∗^*P* < .05. AVJ = atrioventricular junction; Ca_v_1.3 = L-type calcium channel; CN = compact node; His = His bundle; PPB = proximal penetrating bundle; RyR2 = ryanodine 2.

**Supplemental Figure 11:**
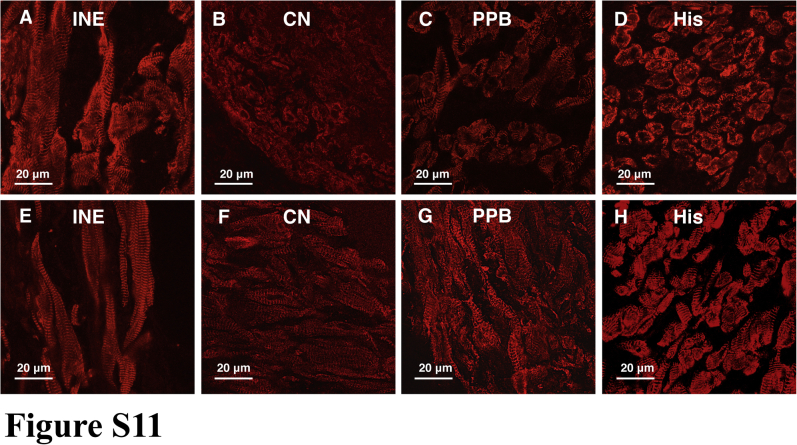
Confocal microscope high magnification images of the AVJ with SERCA2a (red) immunolabelling. Inferior nodal extension (INE), compact node (CN), proximal penetrating bundle (PPB), distal penetrating bundle/His bundle (His). Top panel shows the AVJ components in young hearts and bottom panel shows the AVJ components in old hearts. Bar=20μm.

**Supplemental Figure 12:**
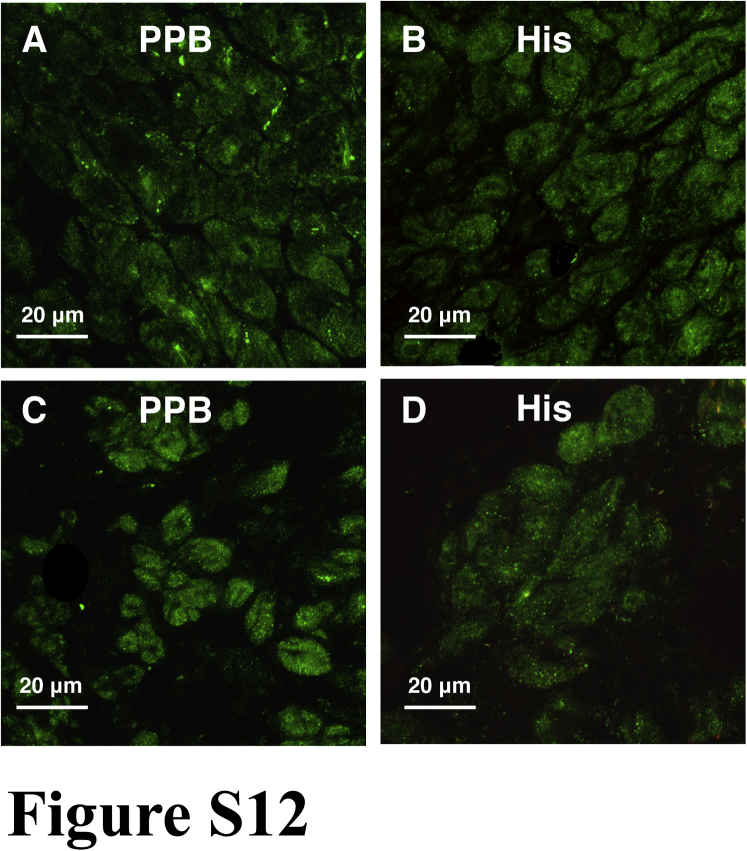
Confocal microscope high magnification images of the AVJ with Cav1.3 (green) immunolabelling, Proximal penetrating bundle (PPB), distal penetrating bundle/His bundle (His). Top panel shows the AVJ components in young hearts and bottom panel shows the AVJ components in old hearts. Bar=20 μm.

We have measured the protein expression of calcium handling proteins including RyR2, sarco/endoplasmic reticulum calcium ATPase (SERCA2a), and Ca_v_1.3 (L-type calcium channel) in all regions of the AVJ (Figure 6). RyR2 expression downregulates by 38% (95% CI −42.67 to −4.13; *P* < .05) with aging in the CN and by 19% (95% CI −13.53 to −0.29; *P* < .05) in the PPB. SERCA2a expression upregulates by 35% (95% CI 7.32–16.67; *P* < .001) with aging in the PPB (Figure 6 and Supplemental Figure S11). Ca_v_1.3 also upregulates by 119% (95% CI 3.99–16.96; *P* < .005) in the PPB (Figure 6 and Supplemental Figure S12).

## Discussion

Aging is associated with AVN dysfunction. This study confirms that the AH interval, WBCL, AVNERP, and AVFRP are all prolonged with aging. This study has yielded important results concerning the cellular mechanisms of AVN conduction and its remodeling with aging. We have demonstrated the effect of I_f_ and RyR2-mediated calcium release from the SR on AVN conduction and changes with aging.

The role of I_f_ and RyR2 in the automaticity of the SN is well established.^7^ However, the evidence for their role in AVN conduction and their association with aging was lacking. Previous studies have analyzed the automaticity of the AVN. Studies in mice show that blocking I_f_ reduces AVN automaticity.^16^, ^18^ The study on AVN conduction in pigs shows that blocking I_f_ with modulating I_Na_ can reduce conduction to a greater degree than blocking these currents individually.^19^ Another study shows that blocking I_f_ can prolong the AH interval in sinus rhythm as well as reduce the ventricular rate during atrial fibrillation in pigs.^20^ Mesirca et al^21^ show that blocking I_f_ can result in AV block. These experiments suggest the role of I_f_ in AVN conduction. Our study confirmed that blocking I_f_ results in prolongation of the AH interval, WBCL, and AVNERP in young rats.

With regard to RyR2, in studies affecting calcium dynamics, a change in AVN automaticity was observed. One study in mice shows that the inhibition of RyR results in decreased automaticity of the AVN.^22^ Another study in canines showed that ryanodine reduces AVJ automaticity by 83%.^23^ Furthermore, blocking L-type calcium channels with isradipine can decrease AVN automaticity.^18^ It is possible that the release of calcium from the SR depends on calcium influx via L-type calcium channels and both mechanisms play a role in AVN conduction. Hancox et al^24^ actually showed that in AVN cells, the calcium influx via L-type calcium channels stimulate calcium release from the SR. Ridely et al^25^ showed that ryanodine (1 μM) decreased the rate of rise of calcium “ramp” and calcium “sparks” that precede calcium transient/influx. These effects, in turn, reduce the rate of rise and amplitude of calcium transient/influx. It is also shown that blocking Na^+^-Ca^2+^ exchanger (NCX), Ca^2+^ current, and SERCA2a reduces spontaneous activity and calcium influx in AVN cells.^26^ Our study confirms that blocking RyR2 results in impaired AVN conduction, prolonging AH interval and WBCL in both young and old hearts. The AH interval is prolonged by 39% in old hearts as compared with 25% in young hearts, which suggests an increased sensitivity to ryanodine with aging.

The increased sensitivity to ryanodine with aging together with almost no effect of blocking I_f_ suggests that the aging AVN is more dependent on calcium dynamics for AVN conduction. This suggests the role of background excitability or depolarization reserve of the nodal cells in AVN conduction. Does less background excitability in the cardiac conduction system result in decreased conduction? The AVN is unique in that aspect. It is more excitable (more automaticity) than bundle branches and Purkinje cells as well as delays. The decrease in automaticity by blocking I_f_ and RyR2 was shown in previous studies.^18^, ^19^, ^20^, ^21^, ^22^, ^23^, ^24^, ^25^, ^26^ By using the same drugs employed to block I_f_ and RyR2 to block the calcium release, we can delay conduction as shown in our functional experiments.

The probable explanation of the decreased automaticity is the increase slope of phase 4 depolarization (via hyperpolarizing membrane current). This may affect AP propagation, as the successive cells need more time to reach the excitation threshold to generate the AP, which ultimately increases the conduction time.

Our immunohistochemical experiments showed reduced expression of RyR2 with aging. RyR2 expression downregulates in the CN and PPB, whereas SERCA2a expression upregulates in the PPB. The net effect of these is possibly reducing the cytosolic calcium concentration. This could result in impaired AVN conduction seen with aging. Tellez et al^27^ showed a substantial decrease in RyR2 expression with aging in the SN, whereas our study correlates with this finding in the AVN. Ca_v_1.3 expression in the PPB increases with aging in our study. This effect will increase the cytosolic calcium concentration, which tends to improve conduction. This increase may well offset the effect of the increased SERCA2a expression in the PPB.

The gap junctions play an important role in cell-to-cell communication. Six connexin proteins combine to form connexons, which, when docked to the connexons of the other cell, form a gap junction channel.^28^ Connexons can be homomeric (connexins of the same type) or heteromeric (different connexins forming connexons). The major isoforms of the connexin family that are expressed in the heart are Cx40, Cx43, Cx45, and Cx30.2. Cx43 is poorly expressed in the SN, INE, and CN, but it is present in the PB. Cx40 is expressed in the AVJ including the INE, CN, and PB. Cx45 is the major gap junction in the INE and CN.^29^, ^30^

Our findings of increased Cx40 in the INE and CN do not support prolonged AVN conduction seen with aging. Cx40 is the largest conductance channel, and thus it allows rapid conduction. It is possible though that Cx40 is coexpressed (heteromeric association) with other connexions. Gemel et al^31^ demonstrated that if Cx40 is coexpressed with Cx30.2, Cx30.2 dominates the voltage-dependent gating, resulting in decreased conduction. Our findings of decreased Cx43 expression in the PPB correlates with findings of slow conduction with aging, as the decreased Cx43 expression (medium conductance channel) will prolong conduction. It correlates with the findings of other studies performed on the aging SN.^32^, ^33^

Age-related downregulation of Na_v_1.5 in the CN and PPB correlates with the study in heterozygous-deficient *SCN5a*/Na_v_1.5 mice. It shows prolonged PR interval and AVNERP.^34^

For the first time, this study has reported a downregulation of HCN4 (His), Na_v_1.5 (CN and PPB), RyR2 (CN and PPB), and Cx43 (PPB and His) with aging whereas the upregulation of SERCA2a (CN and PPB), Ca_v_1.3 (His), and Cx40 (INE and CN) is seen. These findings can explain the functional changes with aging. Increased fibrosis; decreased Cx43, HCN4, RyR2, and Na_v_1.5; and increased SERCA2a are likely to slow conduction. Perhaps, the increased expression of Cx40 and Ca_v_1.3 is a compensatory mechanism. Finally, the increase in the size/volume of the AVJ regions with aging may also contribute to prolonging AVN conduction, but unlikely solely responsible for it. The cellular disarray, hypertrophy, and increase in total collagen content point toward a disruption in cellular architecture. This can also contribute to prolonging AVN conduction.

**Figure 7:**
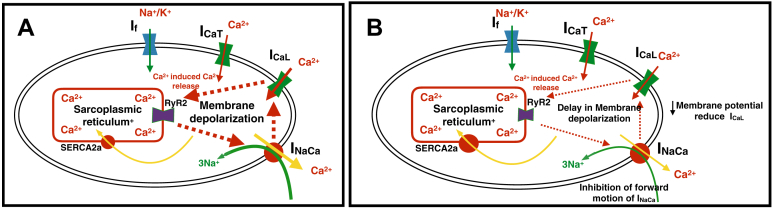
Effect of reduced RyR2 expression with aging on membrane depolarization. **A:** The young atrioventricular nodal myocyte. **B:** The old atrioventricular nodal myocyte. The reduced RyR2 expression with aging results in decreased calcium sparks inhibiting the forward mode of the Na^+^-Ca^2+^ exchanger, reducing inward I_Na-Ca_. This will delay the diastolic membrane potential increase (toward positive membrane potential), thus inhibiting the activation of the L-type calcium channel. I_CaL_ or I_CaT_ = Ca^2+^ current; I_f_ = funny current; I_Na_ = Na^+^ current; I_NaCa_ = sodium-calcium exchange current; RyR2 = ryanodine 2.

Reduced expression of RyR2 and increased sensitivity to ryanodine with aging suggest an important role of RyR2. These changes, we believe, are the most important changes that affect AVN conduction. The reduced oscillatory release of calcium inhibits the forward mode of the NCX, which, in turn, reduced inward sodium calcium exchange current (I_Na-Ca_). This reduced inward I_Na-Ca_ delays the diastolic depolarization, thus inhibiting the activation of the L-type calcium channel. The successive nodal cells thus require more time to reach the excitation threshold. This effect results in delaying conduction across the AVN, as AP propagation is impaired (Figure 7).

### Study limitations

There are few limitations in our study. We have tried multiple commercially available antibodies for K^+^ channels, HCN2, and Cx45, but were unable to get the specific signals required for the correct interpretation. Furthermore, the effect of ryanodine is complex. It has been shown that ryanodine acts as a functional blocker of RyR. The effect of ryanodine on the cardiac SR reduces the calcium handling ability of the SR by forming a long-lasting subconductance state of the calcium release channel. This results in SR calcium depletion, which may increase the basal cytosolic calcium concentration. In contrast, cardiac myocytes can maintain the intracellular calcium content by the NCX.^35^ The increased SERCA2a expression in the PPB can potentially have the consequence of increasing SR calcium, and thus it may affect the forward mode of the NCX; however, this is unlikely with RyR2 changes. We have also not measured NCX expression, which can affect AVN conduction. It is however difficult to predict the effect of reduced or increased NCX expression on AVN conduction without single-cell studies (because of the forward or reverse mode of the NCX in response to intracellular/SR calcium concentration). The future work on single-cell studies on aging AVN cells will likely explain this puzzle.

## Conclusion

From the data presented in this study, we conclude that AVN dysfunction with aging in rats is the result of remodeling of ion channels, connexins, and calcium handling proteins, together with fibrosis and cellular disarray. This remodeling may further worsen in a disease state, resulting in high degree of AV block. We have also shown that I_f_ and RyR2 play a role in AVN conduction. Changes in both these with aging, especially RyR2, are likely to prolong AVN conduction. Studies using a single-cell patch-clamp technique in the aging AVJ to explore the role of I_f_ and RyR2 could further improve our understanding of AVN conduction with aging.

## Figures and Tables

**Supplemental Figure 2A:**
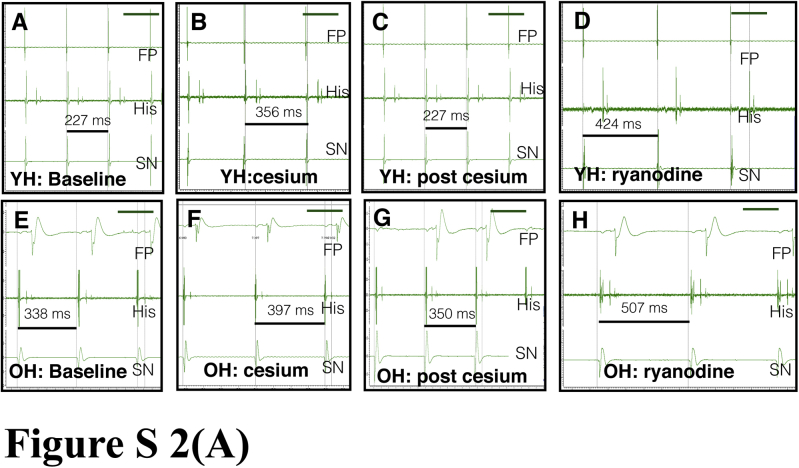
Original recordings showing Sinus node cycle length measurements in Young heart’s (YH) AV nodal preparation and Old heart’s (OH). Recordings are shown at baseline, with caesium (2 mM), after caesium washout and with ryanodine (2uM). The approximate bipolar electrode position are labelled (FP: Fast pathway, SN: Sinus node, His: His bundle. Bar (green)=200 ms (milliseconds).

**Supplemental Figure 2B:**
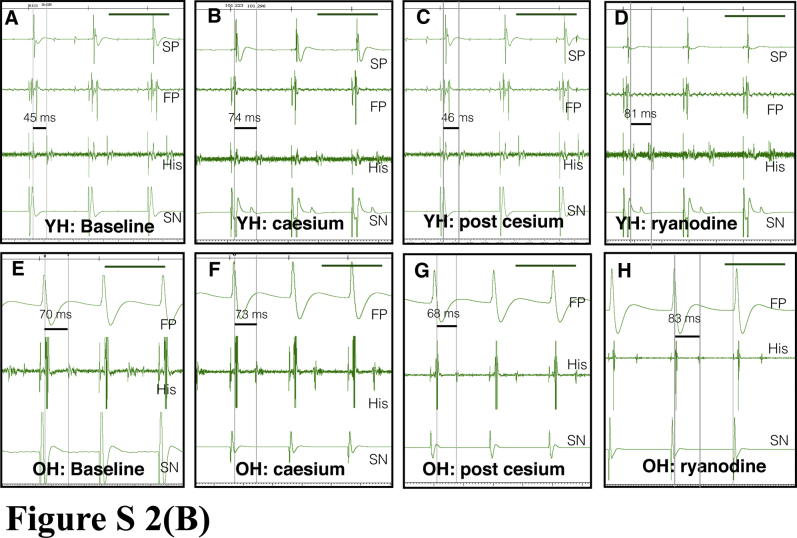
Original recordings showing AH interval measurements in Young heart’s (YH) AV nodal preparation and Old heart’s (OH). Recordings are shown at baseline, with caesium (2 mM), after caesium washout and with ryanodine (2uM). The approximate bipolar electrode position are labelled (FP: Fast pathway, SN: Sinus node, His: His bundle). Bar (green)=200 msec.

**Supplemental Figure 2C:**
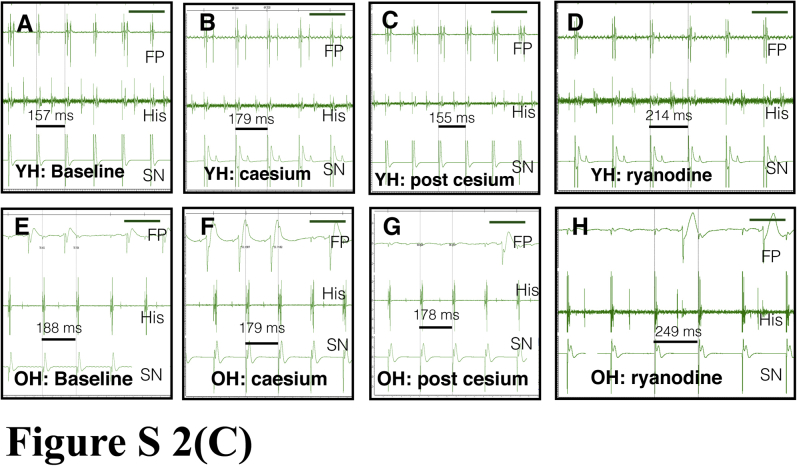
Original recordings showing Wenkebach cycle length measurements in Young heart’s (YH) AV nodal preparation and Old heart’s (OH). Recordings are shown at baseline, with caesium (2 mM), after caesium washout and with ryanodine (2uM). The approximate bipolar electrode position are labelled (FP: Fast pathway, SN: Sinus node, His: His bundle. Bar (green)=200 ms (milliseconds).

**Supplemental Figure 2D:**
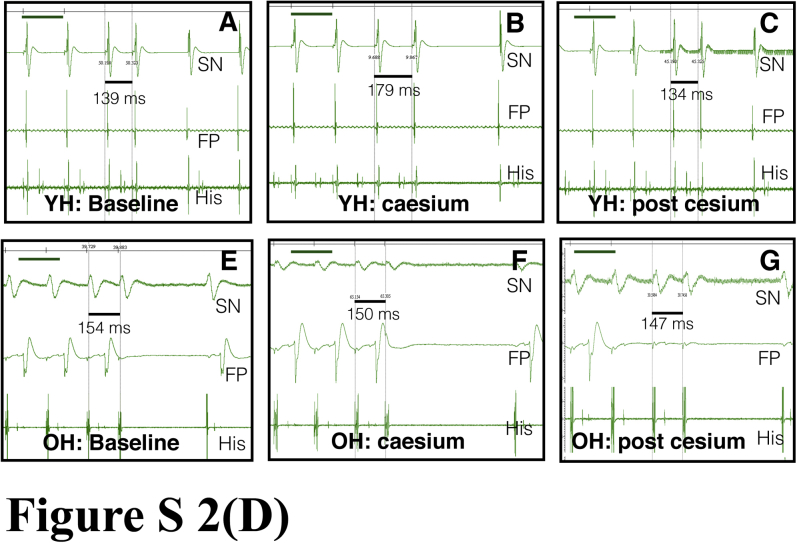
Original recordings showing AV nodal effective refractory period (AVNERP) measurements in Young heart’s (YH) AV nodal preparation and Old heart’s (OH). Recordings are shown at baseline, with caesium (2 mM), after caesium washout and with ryanodine (2uM). The approximate bipolar electrode positions are labelled (FP: Fast pathway, SN: Sinus node, His: His bundle. Bar (green)=200 ms (milliseconds). AVNERP with ryanodine is not measured as the S1 drive train at 200 ms results in Wenkebach phenomenon.
