# Supplementary material for: Structural and functional remodeling of the atrioventricular node with aging in rats: The role of hyperpolarization-activated cyclic nucleotide–gated and ryanodine 2 channels
Source: Heart Rhythm. 2018 May;15(5):752–60. doi: 10.1016/j.hrthm.2017.12.027 (PMC5934612; doi:10.1016/j.hrthm.2017.12.027)
Supplement: Supplemental Material [file mmc1.docx]

# Data supplement

# Materials and Method

## Animals

Male Wistar-Hanover rats were used in this study (Charles River UK Ltd, Kent, UK, Young rats 3 months old and old rats 24 months old). In total, 24 young rats and 15 old rats were used in the study. All animal procedures were performed in accordance with UK animals scientific procedure act 1986. On the survival curves, a 3 months old rat correspond to a 20 year old human and a 24 months old rat correspond to a 70 year old human. The heart weight, body weight and heart weight/body weight ratio are shown in Figure S1.

## Solutions

For electrophysiological experiments, Tyrode’s solution (TS) (NaCl 120 mM, CaCl 1.2 mM, KCl 4mM, MgS0_4_.7H_2_0 1.3 mM, NaH_2_P0_4_.2H_2_O 1.2 mM, NaHC0_3_ 25.2 mM, glucose 5.8 mM) was prepared on the day of the experiment and bubbled with 95% O_2_/5% CO_2_.

## Dissection of the AV node/atrial preparation for electrophysiological experiments

Experiments were carried out on AV nodal preparations from young and old rats (see Figure S1). The dissection method has been described before by our group.^12^ 14 young rats and 6 old rats were used in electrophysiology experiments. Animals were given 1000 I.U of heparin 10-15 minutes before the procedure to prevent coagulation during dissection. Humane euthanasia was performed on these animals via carbon dioxide inhalation and swift dislocation of neck. The chest was opened rapidly, the heart removed, placed and dissected in oxygenated ice-cold TS at 4^0^C. The lung, trachea and excessive connective tissue were removed. The lower part of the ventricles and the superior portion of the left atrium were then removed. The lateral wall of the left ventricle was then cut across up until the left atrium and the whole preparation was then opened up. The RV free wall was then dissected into the right atrium just to the right of the ridge in the right atrial appendage. We have then dissected up to the superior vena cava. The right atrium was then opened and pinned out. Finally the RVOT was opened to expose the AV node. The whole preparation was then pinned on a black silicon and transferred to a tray with the TS at room temperature and then transferred on to a tissue/water bath for the electrophysiological recordings. The preparation was then allowed to recover after the trauma of dissection for a period of 30 mins. The tissue bath was perfused with oxygenated TS at a rate of 20-25 ml/min, the temperature was maintained at 36-37^0^C and pH at 7.35-7.45.

## Electrical recordings

After dissection the tissue preparation was allowed to recover for 30 mins before measuring the recordings. We have measured the spontaneous sinus node (SN) cycle length (SCL), paced AH interval, WB cycle length, AVERP and AVFRP by using bipolar electrodes. The extracellular signals were measured with 4 modified bipolar electrodes, they were made using 0.25 mm silver wire coated with 0.04 mm Teflon (Advent Research Materials, Oxford). The bipolar electrodes were positioned at the site of earliest activation (SN), the atrial septum, at the His bundle and between the coronary sinus and the tricuspid valve annulus. The bipolar electrodes were connected via a headstage (NL100AK, Digitimer, UK) to an amplifier (NL104A, Digitimer, UK) with a gain of 5000 and filtered between 50 – 500 Hz (NL125/6, Digitimer, UK). The amplified and filtered signal was then converted to a digital signal using a data acquisition unit (Micro 1401, Cambridge Electronic Design, UK). It was recorded and analyzed using the Spike2 software (Cambridge Electronic Design, UK). The preparation was then stimulated at the right atrium with a bipolar pacing electrode connected to a DS2A Isolated Constant Voltage Stimulator (Digitimer, UK). The voltage stimulator was connected to the computer via the data acquisition unit, which allowed pacing protocols programmed in Spike 2 to be executed.

The SN cycle length was spontaneously measured. Threshold, AH interval and WB cycle length was determined using a S1-S1 protocol. The atrium was paced with a fixed cycle length for 30 secs starting at 200ms. The cycle length at which 1:1 atrial to ventricular conduction failed and WB conduction occurred using 1 millisecond (ms) reduction in cycle length was recorded as the WB cycle length. AVNERP was determined using a S1-S2 protocol. There was an initial drive train of S1 beats (S1-S1, 180 ms) for 8 beats. The initial S1-S2 coupling interval was 170 ms. The greatest S1-S2 coupling interval that did not conduct after a 1 ms decrement was defined as the AVERP. AVFRP was determined using the protocol described for AVERP. The RR interval between the QRS of the last of the paced beats in the drive train and the QRS elicited by the S2 stimulus, i.e. the R1-R2 interval, was measured and plotted as an AV node conduction curve. The lowest R-R interval represents the fastest conduction across the AV node and was defined as the AVFRP.

Cesium was infused for 20 minutes to reach a steady state concentration before repeating the measurements. Cesium blocks HCN-channels in a voltage dependent manner and the concentration of 2mM is specific for ***I_f_***.^13^ Cesium was then washed off with fresh oxygenated TS until all the recordings reach the pre-cesium level. Ryanodine (2μM) is then infused for 20 minutes before performing the final measurements. In micromolar concentration (2μM), ryanodine block the release of Ca^2+^ from SR.^14^

## Drugs

Cesium Chloride (Sigma Aldrich, Poole, UK) was used in 2mM concentration to block the funny current - ***I_f_*** (HCN channels). Ryanodine (Sigma Aldrich, Poole, UK) was used in 2uM concentration to block RyR channels (block Ca^2+^ release from the sarcoplasmic reticulum).

## Histology

20m tissue sections were stained with Masson’s trichrome (MT) for studying cellular architecture and a picrosirius red (PSR) stain was used for collagen signal estimation. MT connective tissue was stained royal blue, cardiac myocytes are stained pink and nuclei are stained dark blue/black. Images of tissue sections were obtained using a Zeiss DC camera mounted on a Zeiss dissection microscope. PSR staining was used in conjunction with polarized microscopy to estimate collagen signal intensity. Under the polarized microscope large collagen fibers appear red and thin collagen fibers appear green. Volocity software was used to assess collagen signal intensity.

## Immunohistochemistry

Immunohistochemistry was carried out using established methods as described previously on a 20μm tissue sections. Tissue sections were double-labelled with HCN4 and Cx43 to identify the CCS. HCN4 is highly expressed in the CCS, Cx43 on the other hand is poorly expressed. All the primary antibodies specificities were confirmed by performing western blot experiments on the left ventricular tissue. Briefly, tissue sections were fixed in 10% formalin (Sigma) for 30 mins and washed with 0.01 M phosphate buffer solution (PBS) three times at 10 min intervals. Sections were then permeabilized by incubating them in PBS containing 0.1% Triton X-100 for 30 mins, after which they were washed with PBS and then blocked with 1% bovine serum albumin in PBS for 1 h at room temperature. Sections were incubated with the primary antibody (diluted in 1% bovine serum albumin, BSA) at 4^0^C overnight, after which they were washed three times with PBS over 30 min. Sections were incubated with secondary antibodies for 1–2 h at room temperature. After washing three times in PBS, coverslips were mounted on the microscope slides and the coverslips were sealed with nail polish. Slides were stored in the dark at 4^0^C. The secondary antibodies contain fluorescent tags such as Cy3 (indocarbocyaninne) and FITC (fluorescein isothiocyanate), which can be visualized using an epifluorescence or confocal laser-scanning microscope. Immunolabelled tissue was viewed with a Zeiss LSM 510 laser scanning confocal microscope equipped with an argon laser, which allowed excitation at a 496 nm wavelength for the detection of FITC and also a krypton laser which allowed excitation at 556nm for the detection of Cy3. All images presented are single optical sections. Volocity software was then used to analyze signal intensity in arbitrary units. Tissue section preparations were double labelled with HCN4 and Cx43 to identify the cardiac conduction system. HCN4 is a marker of the cardiac conduction system and highly expressed in the INE, CN and PB. Cx43 on the other hand is poorly expressed in the cardiac conduction system. All primary antibodies specificity wasconfirmed by performing western blot experiments on the left ventricular tissue. Summary of the antibodies used are in shown in Table 1S (A and B).

**Table 1S (A). Summary of Primary Antibodies**

| Primary Antibody | Labelled protein | Dilution | Supplier |
| --- | --- | --- | --- |
| Mouse anti-RyR2 | RyR2 | 1:50 | Affinity BioReagants lab |
| Mouse anti-caveolin3 | Cav 3.0 | 1:50 | BD Transduction |
| Mouse anti-SERCA 2a | SERCA-2a | 1:200 | Affinity BioReagants lab |
| Rabbit anti-Ca_V_ 1.3 | Ca_V_ 1.3 | 1:50 | Almone lab |
| Rabbit anti-Na_v_ 1.5 | Na_V_ 1.5 | 1:50 | Almone lab |
| Rabbit anti-HCN4 | HCN4 | 1:50 | Almone lab |
| Rabbit anti-HCN1 | HCN1 | 1:50 | Almone lab |
| Goat anti-Cx40 | Cx40 | 1:50 | Santa cruz lab |
| Mouse anti-Cx43 | Cx43 | 1:100 | Millipore lab |
| Goat anti-Cx45 | Cx45 | 1:100 | Millipore lab |

**Table 1S (B). Summary of Secondary Antibodies**

| Secondary Antibody |  | Dilution | Supplier |
| --- | --- | --- | --- |
| Donkey anti-rabbit FITC |  | 1:100 | Millipore lab |
| Donkey anti-mouse Cy3 |  | 1:400 | Millipore lab |
| Donkey anti-goat FITC |  | 1:100 | Abcam lab |

**Table 2S:** **Comparison between young (n=6) and old rat hearts (n=6). Difference in compact node (CN), proximal penetrating bundle (PPB), distal penetrating bundle or His (DPB/His) three-dimensional size (at low, 10X magnification), number of nuclei (at high, 63X magnification)** (Statistically significant results are shown in red with p value <0.05 or <0.01).

|  | Length (antero- posterior axis)  μm | Width  (horizontal axis)  μm | Height  (Vertical axis)  μm | Volume (uL)   \| volume = \| \| 4 \| \| --- \| \|  \| \| 3 \| \| πabc \| \| --- \| --- \| --- \| --- \| --- \| --- \|   where a, b, and c are the lengths of the axes |
| --- | --- | --- | --- | --- | --- | --- | --- | --- | --- | --- |
| Young CN (Mean± SEM) | 555±62 | 558±56 | 198±42  ρ <0.05 | 0.256 uL |
| OldCN (Mean± SEM) | 545 ± 59 | 599±48 | 456 ±131  ρ <0.05 | 0.623 uL |
| Young PPB (Mean±SEM) | 320 ± 16 | 443±37 | 338±49  ρ <0.05 | 0.200 uL |
| Old PPB  (Mean ±SEM) | 340±25 | 451± 28 | 663±116  ρ <0.05 | 0.425 uL |
| Young His (Mean±SEM) | 890±19 | 453±68 | 304±13  ρ <0.05 | 0.513 uL |
| Old His  (Mean±SEM) | 870±58 | 403±18 | 600±66  ρ <0.05 | 0.881 uL |
